# A retrospective study of laparoscopic, robotic-assisted, and open emergent/urgent cholecystectomy based on the PINC AI Healthcare Database 2017–2020

**DOI:** 10.1186/s13017-023-00521-8

**Published:** 2023-11-30

**Authors:** Stephen Campbell, Shih-Hao Lee, Yuki Liu, Sherry M. Wren

**Affiliations:** 1 VA Medical Center, Palo Alto Division, 3801 Miranda Avenue, Palo Alto, CA 94304 USA; 2 Intuitive Surgical, Inc., Sunnyvale, CA USA; 3 Stanford University School of Medicine, Palo Alto, CA USA

## Abstract

**Background:**

Robotic-assisted cholecystectomy (RAC) is becoming increasingly common, but the outcomes of emergent/urgent robotic-assisted cholecystectomies compared to emergent laparoscopic (LC) and open cholecystectomies (OC) remain understudied.

**Methods:**

The PINC AI Healthcare Database was queried to identify adults who underwent emergent or urgent (Em-Ur) cholecystectomy between January 1, 2017, and December 31, 2020. Immediate postoperative and 30-day outcomes were identified including intraoperative complications, transfusion, conversion, postoperative complication, and hospital length of stay. Propensity score matching was done to compare outcomes between Em-Ur robotic-assisted, laparoscopic, and open cholecystectomies Subgroup analyses were performed comparing RAC done with and without fluorescent imaging as well as comparing RAC and LC performed for patients with class 3 obesity (BMI ≥ 40 kg/m^2^).

**Results:**

RAC Em-Ur cholecystectomies are being performed with increasing frequency and is the most utilized modality for patients with class 3 obesity. There was no difference in intraoperative complications (0.3%), bile duct injury (0.2%), or postoperative outcomes between RAC and LC. LC had significantly shorter operating room times (96 min (75,128)) compared to RAC (120 min (90,150)). There was a significant lower rate of conversion to open in RAC (1.9%) relative to LC (3.2%) in both the overall population and the class 3 obesity sub-analysis (RAC-2.6% vs. LC-4.4%). There was no difference in outcomes in robotic-assisted cholecystectomies done with and without fluorescent imaging.

**Conclusions:**

A comparison of propensity score-matched cohorts of emergent/urgent robotic-assisted and laparoscopic cholecystectomy indicates that robotic-assisted cholecystectomy is a safe alternative to laparoscopic cholecystectomy, and that both have superior outcomes to open cholecystectomies.

**Supplementary Information:**

The online version contains supplementary material available at 10.1186/s13017-023-00521-8.

## Background

Laparoscopic cholecystectomy (LC) is one of the most frequently performed general surgical operations in the USA and is considered to be the gold-standard approach compared to robotic-assisted or open approaches [1, 2]. Elective robotic-assisted cholecystectomy (RAC) is commonly introduced to general surgeons early in their adoption of robotics due to its similarities to laparoscopic cholecystectomy [3–5]. With increased elective RAC experience among surgeons, interest in utilizing the robotics platform for emergent/urgent cases has also increased [6–8].

The outcomes of emergent/urgent (Em-Ur) robotic cholecystectomies have not yet been shown to be equivalent to emergent laparoscopic or open cholecystectomy (OC) in large cohort studies. Adoption of RAC for Em-Ur operations, if shown to have a non-inferior safety profile relative to LC, may have multiple benefits including decreased perioperative complications as well as decreased rates of conversion to open cholecystectomy.

The current retrospective observational study of a large US national database examined the perioperative outcomes for emergent and urgent robotic-assisted cholecystectomies, laparoscopic cholecystectomies, and open cholecystectomies. We hypothesized that when compared to Em-Ur laparoscopic cholecystectomies, Em-Ur robotic-assisted cholecystectomies would have shorter operating room times, a lower rate of intraoperative complications (such as common bile duct injury), and a lower rate of conversion to open cholecystectomy.

## Methods

### Data source

The PINC AI Healthcare Database (PHD, previously known as Premier Healthcare Database) was used for the current study [9]. The PHD is a large US hospital-based Health Insurance Portability and Accountability Act (HIPAA)-compliant database containing inpatient and outpatient data from diverse hospitals and healthcare systems. It includes more than 135 million inpatient admissions, representing 25% of annual US inpatient admissions. The database includes de-identified patient characteristics, treating hospitals and physicians, and billed services, such as medications and devices used during hospitalizations. In the USA, retrospective analyses of the Premier Healthcare Database data are considered exempt from informed consent and institutional review board (IRB) approval pursuant to 45 CFR 46.101(b)(4). The study followed the Strengthening the Reporting of Observational Studies in Epidemiology (STROBE) reporting guideline [10].

### Study population

**Fig. 1:**
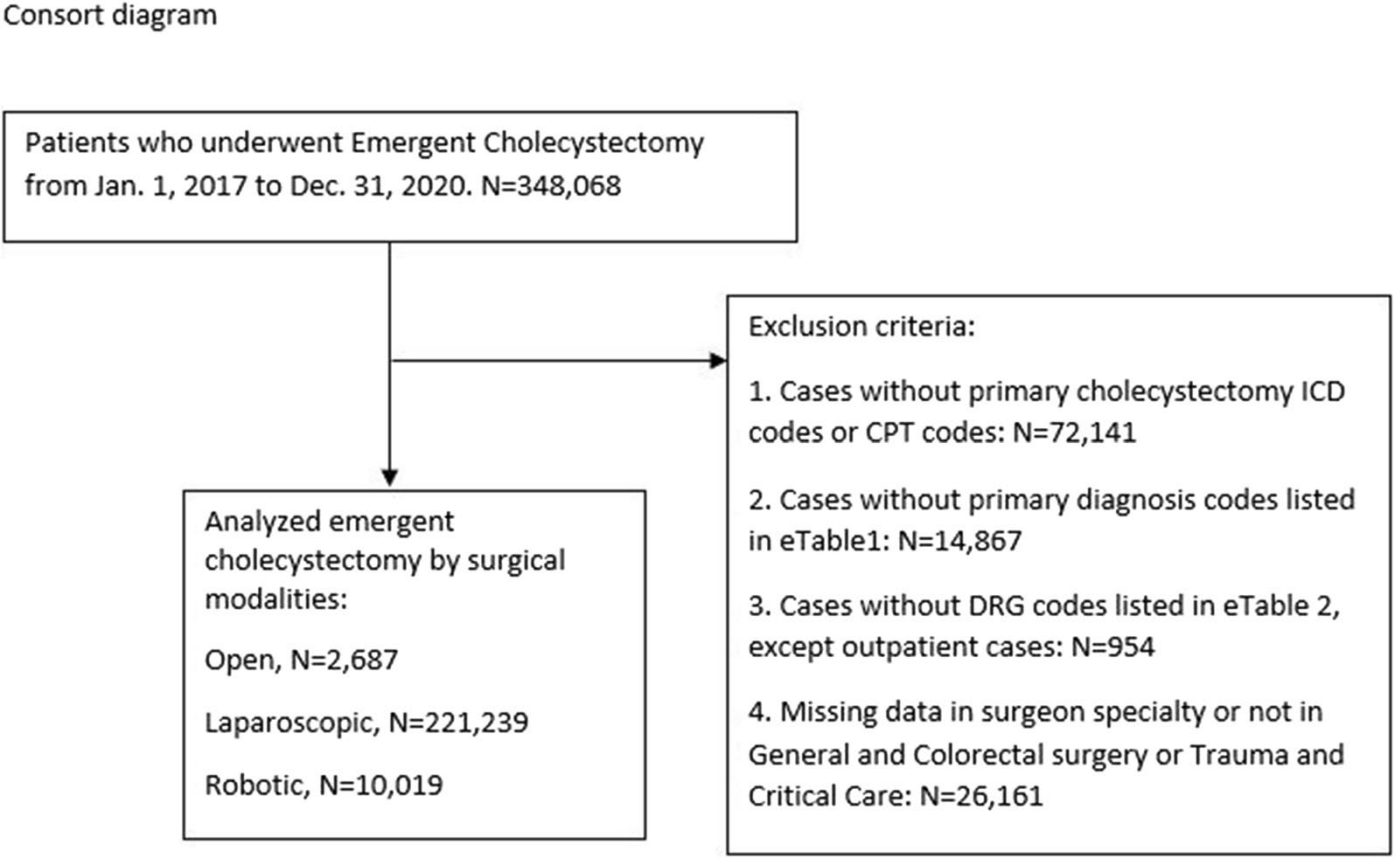
Study selection of patients undergoing emergent cholecystectomy

The study population included adult patients (aged 18 years or older) who underwent emergent or urgent cholecystectomy between January 1, 2017, and December 31, 2020. The PHD database definition of emergent and urgent is based on the Centers for Medicare and Medicaid Services (CMS) UB-04 admission type and is described as ‘the patient required immediate medical intervention as a result of severe, life threatening or potentially disabling conditions. Generally, the patient was admitted through the emergency room’ for emergent and ‘the patient required immediate attention for the care and treatment of a physical or mental disorder. Generally, the patient was admitted to the first available, suitable accommodation’ for urgent. Cases were excluded if: cholecystectomy was not the primary procedure, cases with primary diagnosis codes were not listed in gangrene and perforation, common bile duct stones and disease, cholecystitis without common bile duct stones, biliary pancreatitis, sepsis, and bacteremia (See Additional file 6: eTable 1), diagnosis-related group (DRG) codes did not fall within 411–419, 853–855 (See Additional file 7: eTable 2), and data were missing for surgeon specialty or the specialty was not general, colorectal surgery, or trauma and critical care (Fig. 1). International Classification of Diseases Tenth revision, Clinical Modification (ICD-10-CM) procedure codes, Current Procedure Terminology (CPT) codes, and hospital billing were used to define surgical modalities (Additional file 6: eTable 1). Robotic cholecystectomy was defined as minimal invasive surgery with robotic codes or cases utilizing robotic supplies in hospital billing (Additional file 6: eTable 1). This use of text string search in PHD billing data has been previously validated for the identification of robotic-assisted procedures [11, 12].

### Surgical outcomes and healthcare resource utilization

ICD-10-CM codes were used to assess surgical outcomes such as intraoperative complications, conversion to open surgery, blood transfusion, and postoperative complications (including sepsis, gastrointestinal, digestive, pulmonary, cardiovascular, genitourinary, and wound/infection complications) during index hospitalization and within postoperative 30 days (See Additional file 8: eTable 3). Hospital length of stay (LOS) was calculated as the number of days from admission to discharge. Operating room time was determined from the total time billed for the operating room. Readmissions and reoperations within 30 days were measured by any readmission or scheduled operating room time, respectively, from discharge to the 30-day follow-up.

### Patient and provider characteristics

Patient socio-demographic characteristics included age, gender, race, ethnicity, and insurance type (Medicare, Medicaid, commercial, or others). Patient clinical characteristics included admission type (inpatient, outpatient, outpatient observation), year of admission, primary diagnosis, obesity status, and Charlson Comorbidity Index (CCI) score. Provider characteristics included geographical region (Midwest, Northwest, South, or West), hospital type (community or teaching), location (rural or urban), and bed size (< 200, 200–400, 401–600, or > 600). Physician specialty was classified as general and colorectal surgery, or trauma and critical care. Surgeon volume was calculated individually for each patient and estimated as the number of total cholecystectomies by any operative approach performed by the surgeon during the 12 months before the index procedure in the same hospital [11]. For purposes of analysis, surgeon volume was divided into quartiles: 0–10 procedures were considered low volume, 11–45 low–medium volume, and > 105 high volume [13].

### Exploratory analyses

Multivariate logistic regression analyses were performed on the entire Em-Ur cholecystectomy study population to examine the relationship between independent variables such as surgical modalities and the selected outcome complication variables. Patients were assessed for demographic data, operative indications, clinical characteristics, type of cholecystectomy, and postoperative complications. Altogether, we analyzed 14 potential risk factors to identify any significant variables that influence on the incidence of duct injury, conversion to open approach, and overall perioperative-30-day complications (see Additional file 11: eTable 6–Additional file 13: eTable 8).

### Statistical analysis

Propensity score matching (PSM) [14] was performed to compare outcomes across different surgical modalities given the highly imbalanced cohort groups, large number of cofounders, and rarity of the adverse events. Multivariable logistic regression models were used to generate propensity scores, indicating the likelihood of patients receiving different modalities. Variables to derive propensity scores included patient socio-characteristics, patient clinical characteristics, provider characteristics, physician specialties, physician volume, and key risk factors from exploratory regression analyses. The Greedy 1-to-1 matching algorithm without replacement was used to generate the matched study samples [15]. Covariate balance was evaluated using standardized differences with a threshold of less than 0.1 indicating a negligible difference [16]. Covariate balance plots were generated for both primary and subgroup analyses to visualize the balance of covariates in our analysis (see Additional file 1: eFigure 1–Additional file 5: eFigure 5).

Binary outcomes were analyzed using logistic regression; continuous outcomes were examined using gamma regression. Covariates with standardized differences > 0.1 were further included in post-PSM model for residual covariate imbalance [15].

For the subgroup analysis of fluorescent imaging use in robotic-assisted cholecystectomy, robotic cases were further categorized into those with fluorescent imaging and those without any intraoperative cholangiogram (IOC). IOC was determined using ICD and CPT codes related to radiology diagnosis. Fluorescent imaging was recognized when both the IOC code and hospital billing for indocyanine green were present. Robotic cases with IOC were excluded from the cohort. PSM and regression models were applied to compare surgical outcomes between two groups.

Subgroup analysis also was conducted on patients with class 3 obesity (BMI ≥ 40 kg/m^2^), using PSM and regression models to compare outcomes between groups. Two-sided tests with *p* < 0.05 were deemed statistically significant. All analyses were performed using R version 4.2.1 (R Foundation for Statistical Computing).

## Results

Demographic, clinical, and operative characteristics as well as admission, hospital, and surgeon characteristics prior to matching are summarized in Table 1. From January 1, 2017, to December 31, 2020, a total of 233,945 patients were identified in the dataset as having undergone Ur-Em cholecystectomy: open (*n* = 2687), robotic (*n* = 10,019), or laparoscopic cholecystectomy (*n* = 221,239). Patients were evenly distributed over the age groups of 18–44 years (35%), 45–64 years (33%), and ≥ 65 years (32%). Patients were similar in terms of body mass index (BMI), race, ethnicity, primary diagnosis, and comorbidity burden.

**Table 1:** Comparative patient demographics, medical presentation, hospital, and surgeon characteristics prior to propensity score matching (PSM)^a^

Parameter	Overall (*n* = 233,945)	RAC (*n* = 10,019)	Open (*n* = 2687)	Laparoscopic (*n* = 221,239)
Age groups, *n* (%)				
18–44 years	82,449 (35.0)	3701 (37.0)	379 (14.0)	78,369 (35.0)
45–64 years	76,079 (33.0)	3272 (33.0)	852 (32.0)	71,955 (33.0)
65+ years	75,417 (32.0)	3046 (30.0)	1456 (54.0)	70,915 (32.0)
Sex, *n* (%)				
Female	143,136 (61.0)	6172 (62.0)	1261 (47.0)	135,703 (61.0)
Male	90,809 (39.0)	3847 (38.0)	1426 (53.0)	85,536 (39.0)
Obesity, *n* (%)				
BMI < 30, kg/m^2^	4618 (2.0)	185 (1.8)	187 (7.0)	4246 (1.9)
BMI 30–34, kg/m^2^	17,532 (7.5)	882 (8.8)	178 (6.6)	16,472 (7.5)
BMI 35–39, kg/m^2^	15,662 (6.7)	786 (7.8)	138 (5.1)	14,738 (6.7)
BMI ≥ 40, kg/m^2^	22,014 (9.4)	1166 (12.0)	243 (9.0)	20,605 (9.3)
BMI unknown	174,119 (74.0)	7000 (70.0)	1941 (72.0)	165,178 (75.0)
Ethnicity, *n* (%)				
Hispanic or Latino	37,231 (16.0)	1960 (20.0)	282 (10.0)	34,989 (16.0)
Not Hispanic or Latino	152,365 (65.0)	6311 (63.0)	1945 (72.0)	144,109 (65.0)
Unknown	44,349 (19.0)	1748 (17.0)	460 (17.0)	42,141 (19.0)
Race, *n* (%)				
Caucasian	169,904 (73.0)	7354 (73.0)	1988 (74.0)	160,552 (73.0)
Black	22,393 (9.6)	889 (8.9)	277 (10.0)	21,227 (9.6)
Other	27,186 (12.0)	1263 (13.0)	255 (9.5)	25,668 (12.0)
Unknown	14,462 (6.2)	513 (5.1)	157 (5.8)	13,792 (6.2)
Primary diagnosis category, *n* (%)				
Gangrene and perforation	107 (< 0.1)	8 (< 0.1)	2 (< 0.1)	97 (< 0.1)
CBD stones and disease	41,358 (18.0)	1719 (17.0)	403 (15.0)	39,236 (18.0)
Cholecystitis w/o CBD stones	152,139 (65.0)	6484 (65.0)	1363 (51.0)	144,292 (65.0)
Biliary pancreatitis	21,161 (9.0)	1007 (10.0)	129 (4.8)	20,025 (9.1)
Bacteremia/sepsis	19,180 (8.2)	801 (8.0)	790 (29.0)	17,589 (8.0)
Charlson Comorbidity Score, *n* (%)				
CCI = 0	127,394 (54.0)	5184 (52.0)	955 (36.0)	121,255 (55.0)
CCI = 1	56,902 (24.0)	2605 (26.0)	624 (23.0)	53,673 (24.0)
CCI ≥ 2	49,649 (21.0)	2330 (22.0)	1108 (41.0)	46,311 (21.0)
Census region, *n* (%)				
Midwest	43,094 (18.0)	1524 (15.0)	488 (18.0)	41,082 (19.0)
Northeast	34,507 (15.0)	467 (6.0)	504 (19.0)	33,436 (15.0)
South	106,318 (45.0)	5296 (53.0)	1252 (47.0)	9970 (45.0)
West	50,026 (21.0)	2632 (26.0)	443 (16.0)	46,951 (21.0)
Admission year, *n* (%)				
2017	62,112 (27.0)	1663 (17.0)	814 (30.0)	59,635 (27.5)
2018	62,018 (27.0)	1938 (19.0)	716 (27.0)	59,364 (27.0)
2019	61,540 (26.0)	2718 (27.0)	655 (24.0)	58,167 (26.0)
2020	48,275 (21.0)	3700 (37.0)	502 (19.0)	44,073 (20.0)
Admission type, *n* (%)				
Inpatient	200,582 (86.0)	8288 (83.0)	2659 (99.0)	189,635 (86.0)
Outpatient	7424 (3.2)	534 (5.3)	4 (0.1)	6886 (3.1)
Outpatient observation < 24 h	25,939 (11.0)	1197 (12.0)	24 (0.9)	24,718 (11.0)
Hospital size, *n* (%)				
0–199 beds	54,166 (23.0)	2058 (21.0)	547 (20.0)	51,561 (23.0)
200–299 beds	44,121 (19.0)	2795 (28.0)	400 (15.0)	40,926 (18.0)
300–399 beds	40,393 (17.0)	1626 (16.0)	521 (19.0)	38,246 (17.0)
400–499 beds	27,816 (12.0)	1009 (10.0)	312 (12.0)	26,495 (12.0)
500+ beds	67,449 (29.0)	2531 (25.0)	907 (34.0)	64,011 (29.0)
Physician specialty, *n* (%)				
General and colorectal surgery	222,103 (95.0)	9679 (96.6)	2480 (92.3)	209,944 (95.0)
Trauma and critical care surgery	11,842 (5.1)	340 (3.4)	207 (7.7)	11,295 (5.0)
Cholecystectomy volume 1 year prior to index surgery, *n* (%)				
High volume group^b^	78,559 (34.0)	4156 (41.0)	727 (27.0)	73,676 (33.0)
Medium volume group^c^	86,947 (37.0)	3765 (38.0)	1074 (40.0)	82,108 (37.0)
Low volume group^d^	68,439 (29.0)	2098 (21.0)	886 (33.0)	65,455 (30.0)

PSM = Propensity score matching; RAC = Robotic-assisted cholecystectomy; SD = Standard deviation of the mean; BMI = Body mass index; CCI = Charlson Comorbidity Index

^a^Covariates for matching were: age, sex, BMI, ethnicity, race, census region, presence of comorbidities, and CCI score

^b^Median number of procedures = 105

^c^Median number of procedures = 45

^d^Median number of procedures = 10

The frequency of robotic cholecystectomy increased each year from 2017 (2.6%) through 2020 (7.7%). Both the incidence of open cholecystectomies and laparoscopic cholecystectomies decreased over the same period (1.3–1% and 96–91.3%, respectively). Sixty-five percent (*n* = 152,139) of cholecystectomies were performed for cholecystitis across all surgical modalities (open, robotic, laparoscopic); the next most common indication for cholecystectomy was choledocholithiasis (18%), followed by biliary pancreatitis (9%). RAC was more commonly performed in urban medical centers (92%) compared to rural medical centers (8%). Hospital size did not impact likelihood of the robotic approach. RACs were least likely to be performed by low volume surgeons (21%).

Fluorescent-guided imaging was performed more frequently in RAC (44.2%) compared to LC (1.4%). Intraoperative cholangiography more commonly was incorporated in LC (9.1%) versus RAC (4.8%). The majority of cases had neither intraoperative cholangiogram nor fluorescent imaging.

### Propensity score analysis

Propensity score analyses was performed comparing RAC and LC (Table 2), RAC and OC (Table 3), and LC to OC (Table 4). For the RAC and LC comparison, there were 9996 matched patients, and overall there were no significant differences between the groups for intraoperative, postoperative complications, or 30-day postoperative outcomes (Table 2). There was a statistically significant decreased rate of conversions in RAC compared to LC (1.9% vs. 3.2%, *p* < 0.001). Mean and median (IQR) operating room times were significantly longer for RAC (128.4 min; 120 (91,150) compared to LC (110.7 min; 96 (75,128) (*p* < 0.001). Median hospital length of stay was identical.

**Table 2:** Robotic-assisted and laparoscopic cholecystectomy outcomes and 30-day post-surgery following post-propensity score matching (PSM)

Parameter	RAC (*n* = 9996)	Laparoscopic (*n* = 9996)	*p*-value
Index hospitalization outcomes			
Intraoperative complications, *n* (%)	27 (0.3)	26 (0.3)	0.88
Postoperative complications, *n* (%)	1158 (12.0)	1074 (11.0)	0.06
Gastrointestinal and digestive complications, *n* (%)			
Bile duct injury	21 (0.2)	16 (0.2)	0.42
Retained gallstone	2 (< 0.1)	7 (< 0.1)	0.23
Intestinal obstruction	186 (1.9)	171 (1.7)	0.46
Gastrointestinal ulcer	11 (0.1)	9 (0.1)	0.74
Gastrointestinal hemorrhage	26 (0.3)	26 (0.3)	> 0.99
Wound infection/complications, *n* (%)			
Surgical site infection	16 (0.2)	18 (0.2)	0.77
Hemorrhage/hematoma/seroma	14 (0.1)	22 (0.2)	0.27
Wound disruption/dehiscence	108 (1.1)	120 (1.3)	0.48
Drainage of intraperitoneal abscess	13 (0.1)	5 (< 0.2)	0.07
Blood transfusion, *n* (%)	197 (2.0)	199 (2.0)	> 0.99
Conversion, *n* (%)	190 (1.9)	317 (3.2)	< 0.001
Pulmonary complications, *n* (%)	350 (3.5)	300 (3.0)	0.05
Cardiovascular complications, *n* (%)	106 (1.1)	88 (0.9)	0.20
Hospital length of stay, d			< 0.001
Mean (SD)	3.6 (3.6)	3.4 (3.7)	
Median (IQR)	3 (1, 4)	3 (1, 4)	
Operating room time, min			< 0.001
Mean (SD)	128.4 (58.5)	110.7 (49.4)	
Median (IQR)	120 (90,150)	96 (75,128)	
30-day postoperative outcomes			
Sepsis/septic shock, *n* (%)	456 (4.6)	400 (4.0)	0.05
Gastrointestinal and digestive complications, *n* (%)			
Bile duct injury	36 (0.4)	30 (0.3)	0.55
Retained gallstone	4 (< 0.1)	9 (< 0.1)	0.22
Intestinal obstruction	198 (2.0)	182 (2.0)	0.42
Gastrointestinal ulcer	26 (0.3)	15 (0.2)	0.09
Gastrointestinal hemorrhage	48 (0.5)	48 (0.5)	> 0.99
Wound/infection complications, *n* (%)			
Surgical site infection	81 (0.8)	78 (0.8)	0.83
Hemorrhage/hematoma/seroma	32 (0.3)	43 (0.4)	0.23
Wound disruption/dehiscence	145 (1.5)	160 (1.6)	0.39
Drainage of intraperitoneal abscess	13 (0.1)	5 (< 0.1)	0.07
Pulmonary complications, *n* (%)	407 (4.1)	361 (3.6)	0.09
Cardiovascular complications, *n* (%)	174 (1.7)	167 (1.7)	0.70
30-day readmission, n (%)^a^	545 (5.5)	484 (4.8)	0.05
30-day reoperation, n (%)^b^	101 (1.9)	93 (0.9)	0.60
Mortality, *n* (%)	54 (0.5)	42 (0.4)	0.20

RAC = Robotic-assisted cholecystectomy; SD = standard deviation of the mean; and IQR = interquartile range

^a^Sepsis, pancreatitis, retained gallstone/biliary obstruction, renal failure, postoperative pain, pulmonary embolism, nausea/vomiting, peritoneal abscess, ileus, aspiration, postoperative seroma. Any readmission from discharge to 30-day follow-up

^b^Retained gallstone, sepsis/infection, incisional hernia/wound disruption, bowel obstruction. Measured from scheduled OR from discharge to 30-day follow-up

**Table 3:** Robotic-assisted and open cholecystectomy outcomes and 30-day post-surgery following post-propensity score matching (PSM)

Parameter	RAC (*n* = 2054)	Open (*n* = 2054)	*p*-value
Index hospitalization outcomes			
Intraoperative complications, *n* (%)	13 (0.6)	24 (1.2)	0.07
Postoperative complications, *n* (%)	386 (19.0)	652 (32.0)	< 0.001
Sepsis, *n* (%)	172 (8.4)	218 (11.0)	0.02
Gastrointestinal and digestive complications, *n* (%)			
Bile duct injury	6 (0.3)	5 (0.2)	0.88
Retained gallstone	0 (0.0)	1 (< 0.1)	> 0.99
Intestinal obstruction	71 (3.5)	155 (7.5)	< 0.001
Gastrointestinal ulcer	3 (0.1)	13 (0.6)	0.03
Gastrointestinal hemorrhage	8 (0.4)	14 (0.7)	0.22
Wound infection/complications, *n* (%)			
Surgical site infection	5 (0.2)	18 (0.9)	0.01
Hemorrhage/hematoma/seroma	7 (0.3)	15 (0.7)	0.09
Wound disruption/dehiscence	23 (1.1)	59 (2.9)	< 0.001
Drainage of intraperitoneal abscess	8 (0.4)	51 (2.5)	< 0.001
Blood transfusion, *n* (%)	88 (4.3)	252 (12.0)	< 0.001
Pulmonary complications, *n* (%)	133 (6.5)	318 (16.0)	< 0.001
Cardiovascular complications, *n* (%)	34 (1.7)	96 (4.7)	< 0.001
Hospital length of stay, d			< 0.001
Mean (SD)	5.0 (4.8)	8.0 (7.2)	
Median (IQR)	4 (2, 6)	6 (4, 10)	
Operating room time, min			< 0.001
Mean (SD)	142.8 (65.8)	161.6 (70.4)	
Median (IQR)	150 (115, 195)	120 (93, 180)	
30-day postoperative outcomes			
Sepsis, *n* (%)	198 (9.6)	261 (13.0)	0.01
Gastrointestinal and digestive complications, *n* (%)			
Bile duct injury	10 (0.5)	15 (0.7)	0.32
Retained gallstone	1 (< 0.1)	1 (< 0.1)	> 0.99
Intestinal obstruction	74 (3.6)	170 (8.3)	< 0.001
Gastrointestinal ulcer	7 (0.3)	16 (0.8)	0.07
Gastrointestinal hemorrhage	14 (0.7)	20 (1.0)	0.30
Wound infection/complications, *n* (%)			
Surgical site infection	18 (0.9)	55 (2.7)	< 0.001
Hemorrhage/hematoma/seroma	13 (0.6)	32 (1.6)	0.01
Wound disruption/dehiscence	40 (1.9)	104 (5.1)	< 0.001
Drainage of intraperitoneal abscess	8 (0.4)	52 (2.5)	< 0.001
Pulmonary complications, *n* (%)	147 (7.2)	331 (16.0)	< 0.001
Cardiovascular complications, *n* (%)	63 (3.1)	121 (5.9)	< 0.001
30-day readmission, *n* (%)^a^	166 (8.1)	212 (10.3)	0.01
30-day reoperation, *n* (%)^b^	37 (1.8)	45 (2.2)	0.41
Mortality, *n* (%)	27 (1.3)	73 (3.8)	< 0.001

RAC = Robotic-assisted cholecystectomy; SD = standard deviation of the mean; and IQR = interquartile range

^a^Sepsis, pancreatitis, retained gallstone/biliary obstruction, renal failure, postoperative pain, pulmonary embolism, nausea/vomiting, peritoneal abscess, ileus, aspiration, postoperative seroma. Any readmission from discharge to 30-day follow-up

^b^Retained gallstone, sepsis/infection, incisional hernia/wound disruption, bowel obstruction. Measured from scheduled OR from discharge to 30-day follow-up

**Table 4:** Laparoscopic and open cholecystectomy outcomes and 30-day post-surgery following post-propensity score matching (PSM)

Parameter	Laparoscopic (*n* = 2637)	Open (*n* = 2637)	*p*-value
Index hospitalization outcomes			
Intraoperative complications, *n* (%)	24 (0.9)	33 (1.3)	0.2
Postoperative complications, *n* (%)	601 (23.0)	932 (35.0)	< 0.001
Sepsis, *n* (%)	257 (9.7)	327 (12.0)	0.01
Gastrointestinal and digestive complications, *n* (%)			
Bile duct injury	8 (0.3)	11 (0.4)	0.55
Retained gallstone	2 (< 0.1)	1 (< 0.1)	0.64
Intestinal obstruction	86 (3.3)	208 (7.9)	< 0.001
Gastrointestinal ulcer	5 (0.2)	20 (0.8)	0.00
Gastrointestinal hemorrhage	16 (0.6)	25 (0.9)	0.24
Wound infection/complications, *n* (%)			
Surgical site infection	3 (0.1)	27 (1.0)	< 0.001
Hemorrhage/hematoma/seroma	9 (0.3)	16 (0.6)	0.26
Wound disruption/dehiscence	27 (1.0)	86 (3.3)	< 0.001
Drainage of intraperitoneal abscess	4 (0.2)	68 (2.6)	< 0.001
Blood transfusion, *n* (%)	147 (5.6)	372 (14.0)	< 0.001
Pulmonary complications, *n* (%)	213 (8.1)	476 (18.0)	< 0.001
Cardiovascular complications, *n* (%)	71 (2.7)	133 (5.0)	< 0.001
Hospital length of stay, d			< 0.001
Mean (SD)	5.3 (4.9)	8.6 (8.0)	
Median (IQR)	4 (2, 7)	6 (4, 11)	
Operating room time, min			< 0.001
Mean (SD)	130.1 (58.9)	166.6 (72.3)	
Median (IQR)	120 (90, 150)	150 (120, 201)	
30-day postoperative outcomes			
Sepsis, *n* (%)	295 (11.0)	394 (15.0)	< 0.001
Gastrointestinal and digestive complications, *n* (%)			
Bile duct injury	13 (0.5)	25 (0.9)	0.06
Retained gallstone	2 (< 0.1)	1 (< 0.1)	0.64
Intestinal obstruction	87 (3.3)	224 (8.5)	< 0.001
Gastrointestinal ulcer	7 (0.3)	26 (1.0)	0.00
Gastrointestinal hemorrhage	23 (0.9)	34 (1.3)	0.15
Wound infection/complications, *n* (%)			
Surgical site infection	18 (0.9)	55 (2.7)	< 0.001
Hemorrhage/hematoma/seroma	13 (0.6)	32 (1.6)	0.01
Wound disruption/dehiscence	40 (1.9)	104 (5.1)	< 0.001
Drainage of intraperitoneal abscess	8 (0.4)	52 (2.5)	< 0.001
Pulmonary complications, *n* (%)	225 (8.5)	503 (19.0)	< 0.001
Cardiovascular complications, *n* (%)	126 (4.8)	169 (6.4)	0.01
30-day readmission, *n* (%)^a^	209 (7.9)	292 (11.1)	< 0.001
30-day reoperation, *n* (%) ^b^	40 (1.5)	60 (2.3)	0.05
Mortality, *n* (%)	50 (1.9)	133 (5.0)	< 0.001

SD = Standard deviation of the mean; IQR = interquartile range

^a^Sepsis, pancreatitis, retained gallstone/biliary obstruction, renal failure, postoperative pain, pulmonary embolism, nausea/vomiting, peritoneal abscess, ileus, aspiration, postoperative seroma. Any readmission from discharge to 30-day follow-up

^b^Retained gallstone, sepsis/infection, incisional hernia/wound disruption, bowel obstruction. Measured from scheduled OR from discharge to 30-day follow-up

There were 2054 matched patients in the RAC compared to OS analysis (Table 3) and 2637 matched patients in the LC compared to OC analysis (Table 4). For both analyses, open cholecystectomy was significantly associated with higher rates of overall postoperative complications, hospital LOS, 30-day mortality, 30-day readmissions, and blood transfusion (Tables 3 and 4). Index hospitalization complications were all significantly greater in the OC cohort except for: bile duct injury, gastrointestinal hemorrhage, and wound hematoma/seroma. Analysis of 30-day outcomes demonstrated significantly lower rates for the both cohorts for complications including: sepsis, intestinal obstruction, all wound infections/complications, pulmonary complications, cardiovascular complications, and drainage of intraperitoneal abscess.

Differences were seen in operating room time which were significantly longer for robotic cholecystectomy [RAC 150 min (115, 195) vs. OC min 120 (93, 180) *p* < 0.001)] and shorter for LC [120 min (90,150) vs. OS 150 min (120,201) *p* < 0.001)].

### Subgroup analyses for cholangiogram and Class III obesity

Subgroup analysis of 3267 propensity-matched patients RAC performed with near-infrared (fluorescent) imaging and RAC performed without either fluorescent imaging or IOC showed no significant difference in intraoperative complications, immediate postoperative complications, median operating room time, and 30-day postoperative outcomes including bile duct injury, retained gallstone, surgical site infection, or 30-day readmission (Additional file 9: eTable 4).

A total of 1133 matched pairs were analyzed for outcomes in RAC or LC in patients with Class 3 obesity (Additional file 10: eTable 5). RAC was associated with a significant lower rate of conversion to OC (2.6%) compared to LC (4.4%, *p* = 0.024). There were no significant differences in intraoperative complications, immediate postoperative complications, postoperative to 30-day outcomes/complications, and readmission between the two operative approaches. Operative time was significantly longer in RAC (120 min (101,172) compared to LC (110 min (90,150) (*p* < 0.001).

### Exploratory analysis for risks for key outcomes (bile duct injury, conversions, complications)

Multivariate regression analysis for significant risk factors associated with bile duct injury was open cholecystectomy (2.15 (1.44–3.21), *p* < 0.001), male sex (1.22 (1.06–1.41), *p* < 0.01), and primary diagnosis of bacteremia/sepsis (1.75 (1.37–2.24), *p* < 0.00) or CBD stones and disease (3.94 (3.38–4.60), *p* < 0.001) (Additional file 11: eTable 6). Conversely, surgery performed in later admission years (2020: 0.50 (0.41–0.63), *p* < 0.001) and outpatient procedures (0.47 (0.24–0.92), *p* < 0.03) were associated with a significantly lower risk of bile duct injury.

Significant lower risks for conversion to OC were seen in patients that had RAC (0.54 (0.47–0.63), *p* < 0.001), a primary diagnosis of biliary pancreatitis (0.42 (0.38–0.47), *p* < 0.001) or common bile duct stone-related disease (0.87 (0.82–0.92), *p* < 0.001), outpatient procedures (outpatient 0.03 (0.01–0.07), *p* < 0.001 and outpatient observation < 24 h. 0.02 (0.01–0.04), *p* < 0.001), and surgeons with a higher volume of cholecystectomy in the year prior to the index surgery [0.77 (0.73–0.82), *p* < 0.001) (Additional file 12: eTable 7).

Significant risk factors for increased postoperative overall complications (Additional file 13: eTable 8) were open cholecystectomy (2.41 (2.19–2.65), *p* < 0.001), male patients (1.20 (1.16–1.24), *p* < 0.001), higher BMIs, any diagnosis other than cholecystitis without common bile duct stone with the greatest risk a diagnosis of bacteremia/sepsis (6.44 (6.20–6.69), *p* < 0.001), CCI score > 0, and outpatient observation < 24 h. (1.95 (1.86–2.06), *p* < 0.001).

### Covariate balance

The covariate balance tests for RAC versus LC, RAC versus OC, LC versus OC as well as RAC with or without fluorescent imaging and RAC versus lap in patients with class 3 obesity indicated that the absolute standardized mean differences were < 0.1 for all covariates indicating a satisfactory covariate balance (see Additional file 1: eFigure 1–Additional file 5: eFigure 5).

## Discussion

The utilization and outcomes of surgical modalities and outcomes of emergent and urgent cholecystectomy in a contemporaneous acute care setting are described in the current study. A propensity-matched comparison of robotic-assisted and laparoscopic cholecystectomy adds important context to current practice patterns in the USA. Our results indicate that there were no significant differences in intraoperative or postoperative complications in comparing RAC to LC. Our findings of equivalent bile duct injury between RAC and LC and overall bile duct injury rate are consistent with previous studies [17, 18]. These findings support the safe use of robotic-assisted cholecystectomies for emergent or urgent indications in the acute setting.

Robotics may offer a potential benefit for the emergent/urgent treatment of cholecystitis as shown by the observed lower rate of conversion to open surgery. These procedures can be challenging with significant inflammation and omental adhesions. Improved visualization and wristed instruments may offer an advantage in cases with severe inflammation or in patients with significant levels of obesity. Our observation of lower rates of conversion in RAC compared to LC has also been observed in other more complicated hepatobiliary procedures [19, 20]. Conversion of an minimally invasive surgery can lead to a cascade of adverse outcomes leading to increased hospital resource utilization. [21]

Not surprisingly the minimally invasive approach, either RAC of LC, showed clear benefit over OC as demonstrated by lower rates of postoperative complications and outcomes. As would be expected, there were only 2687 primary open procedures (1.1%) in the entire dataset. This cohort had the highest rate of bacteremia/sepsis as the primary diagnosis (29%) as well as surgeons who identified as trauma/critical care (7.7%) performing the procedure. The operative decision to perform a planned open cholecystectomy and not a conversion from RAC or LC is typically reserved for a very small subset of patients based on clinical factors where laparotomy may be indicated due to the patient’s condition or comorbid medical diseases. Unfortunately, there is not enough clinical information in the dataset beyond primary diagnosis and procedure codes so it is unclear what drove the decision to perform an open procedure, and this may be a limitation of the comparisons of both RAC and LC to this population even with propensity matching.

The variable and conflicting literature regarding the benefits and safety of emergent robotic-assisted cholecystectomy has contributed to diverse opinions on whether it is a safe and appropriate approach to managing emergent cholecystectomies, despite the increasing adoption in the USA [22]. Studies have established that RAC has similar outcomes to laparoscopic cholecystectomy in the non-emergent setting but called for further investigation into its safety and appropriateness in the acute setting [3, 4, 26]. Reasons for the observed increasing utilization of robotics may include often the cited benefits of wristed instrumentation, camera stability and control, bipolar and monopolar energy options, near-infrared fluorescence imaging, and no physical effort in countering torque forces of large abdominal walls. This study provides additional data to the safety and outcomes for the growing application of the robotic platform to urgent or emergent cholecystectomy procedures. The lower rate of conversions to open surgery may be from improved visualization of critical structures in the setting of significant inflammation, finer and more controlled dissection, and the perception of easier use in patients with additional factors such as obesity or chronic liver disease [23–26]. An unexpected observation was that near-infrared imaging with ICG in both RAC and LC platforms, which has been reported to result in lower rates of bile duct injury and conversion to open surgery, demonstrated no significant difference in either outcome in the RAC cohort which was an unexpected result [16, 24].

This study also supports the ongoing development of formal robotic acute care curricula in residency and fellowship training. These structured curricula can provide trainees with didactic and operative knowledge so they can utilize robotic MIS techniques to potentially improve patient outcomes [27]. One of the challenges of this is overcoming the learning curve associated with robotic surgery with elective cases prior to implementing robotic surgery with acute care surgery patients. Existing curricula being used in training programs attempt to define intraoperative components and help residents progress through graded autonomy with ongoing assessment progress through the curriculum, which is separate from industry curricula which focus on the adoption of robotic surgery by fully trained surgeons [28]. The goal of the curriculum is to help trainees develop the personal confidence and skill set to appropriately employ robotic MIS techniques in emergency surgeries [29].

Despite the dataset representing a substantial portion of surgical care in the US, our study has several limitations. Due to the retrospective design, the possibility of selection bias remains despite incorporation of propensity score matching. The routinely collected data in the selected database also carry a risk of misclassification bias. However, chargemaster data within PINC AI data enable us to more accurately classify surgical modality, particularly laparoscopic or robotic cases compared to claims data analysis, by identifying instrument and accessories unique to robotic cases. Due to variability in coding of robotic add on codes in the USA, claims data underestimate the prevalence of robotic-assisted cases, mis-classifying them as laparoscopic cases. An additional strength of this dataset is our ability to identify conversions from both RAC and LC to open procedures. We were unable to directly compare the outcomes between robotic, laparoscopic, and open cholecystectomies from the same cohort of surgeons, which could show a difference—particularly in operative times. Another limitation of the data set is the inability to determine if a total versus subtotal cholecystectomy was performed. Future studies comparing laparoscopic to robotic cholecystectomy for urgent or emergent indications should not only track conversion rate but also include data on whether a total cholecystectomy was completed.

## Conclusion

This comparison of propensity score-matched cohorts of emergent or urgent cholecystectomy patients whose procedures were performed with robotic-assistance and laparoscopically indicate that robotic-assisted cholecystectomy is a safe alternative to laparoscopic cholecystectomy, and that both have superior outcomes to open cholecystectomies. Robotic-assisted cholecystectomy can be used for emergent cholecystectomies based on surgeon preference/discretion and hospital capability.

### Supplementary Information

**Additional file 1: eFigure 1** Covariate balance analysis comparing robotic-assisted cholecystectomy (RAC) to laparoscopic cholecystectomy (LC).

**Additional file 2: eFigure 2** Covariate balance analysis comparing laparoscopic cholecystectomy (LC) to open cholecystectomy (OC).

**Additional file 3: eFigure 3** Covariate balance analysis comparing robotic-assisted cholecystectomy (RAC) with open cholecystectomy (OC).

**Additional file 4: eFigure 4** Covariate balance analysis comparing robotic-assisted cholecystectomy (RAC) with fluorescent imaging vs RAC without any cholangiogram or fluorescent imaging.

**Additional file 5: eFigure 5** Covariate balance analysis comparing robotic-assisted cholecystectomy (RAC) and laparoscopic cholecystectomy (LC) in patients with class 3 obesity.

**Additional file 6: eTable 1** Diagnosis and Procedure Codes Used to Identify Cases.

**Additional file 7: eTable 2** Diagnosis-Related Group (DRG) Codes Used for exclusion criteria.

**Additional file 8: eTable 3** Complications code list.

**Additional file 9: eTable 4** Subgroup analyses post-propensity score matching (PSM): outcomes following robotic cholecystectomy without vs with fluorescent imaging.

**Additional file 10: eTable 5** Subgroup analyses post-propensity score matching (PSM): outcomes following robotic and laparoscopic cholecystectomy on patients with class III obesity.

**Additional file 11: eTable 6** Multivariate analysis to identify risk factors for bile duct injury.

**Additional file 12: eTable 7** Multivariate analysis to identify risk factors for conversion.

**Additional file 13: eTable 8** Multivariate analysis to identify risk factors for postoperative complication.

## Data Availability

The datasets generated and/or analyzed during the current study are availability in the PINC AI Healthcare Database, https://offers.premierinc.com/rs/381-NBB-525/images/Premier-Healthcare-Database-Whitepaper-Final.pdf.

## Abbreviations

Em-Ur: Emergent/urgent
RAC: Robotic-assisted cholecystectomy
LC: Laparoscopic cholecystectomy
OC: Open cholecystectomy
PSM: Propensity score matching
LOS: Hospital length of stay
CCI: Charlson Comorbidity Index
IOC: Intraoperative cholangiography
