# Supplementary figures and images for: A retrospective study of laparoscopic, robotic-assisted, and open emergent/urgent cholecystectomy based on the PINC AI Healthcare Database 2017–2020
Source: World J Emerg Surg. 2023 Nov 30;18:55. doi: 10.1186/s13017-023-00521-8 (PMC10687827; doi:10.1186/s13017-023-00521-8)

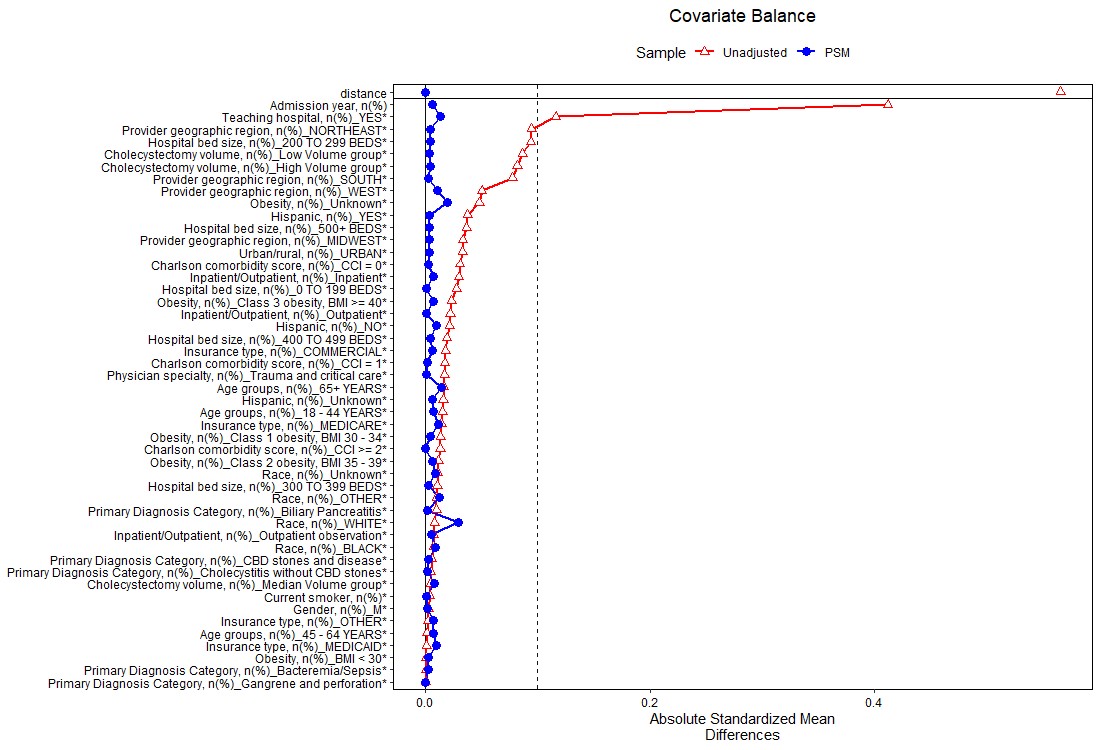

Supplement: Supplementary file 1 — Additional file 1: eFigure 1 Covariate balance analysis comparing robotic-assisted cholecystectomy (RAC) to laparoscopic cholecystectomy (LC). [file 13017_2023_521_MOESM1_ESM.jpg]

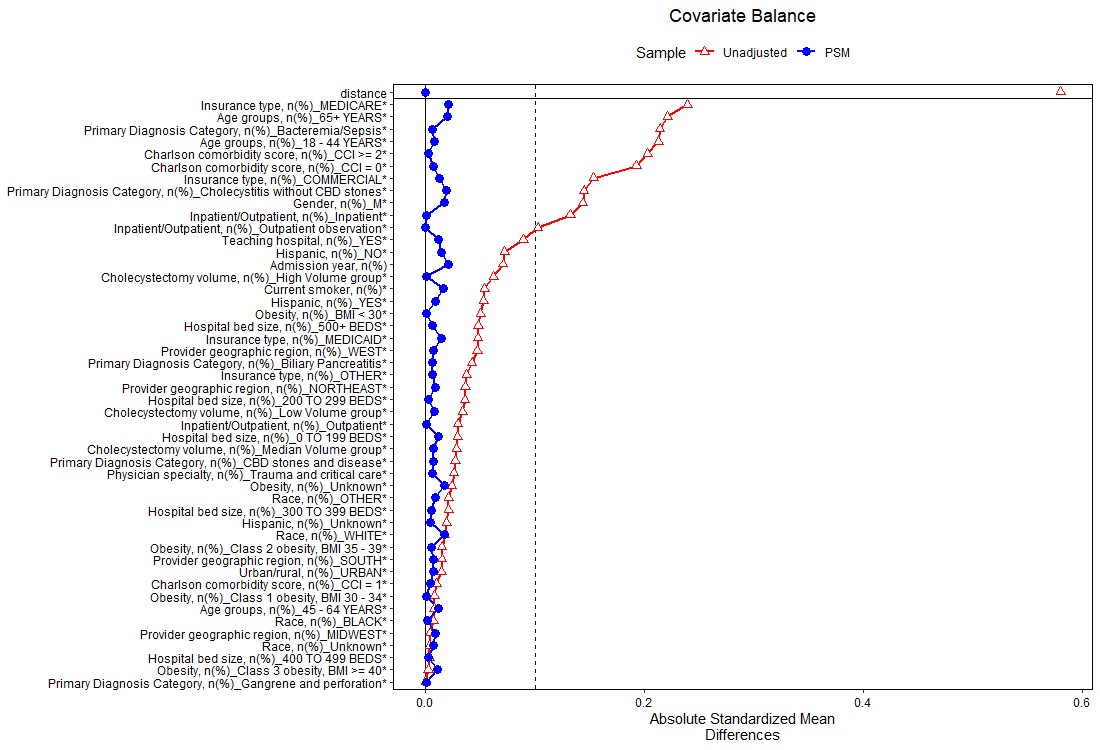

Supplement: Supplementary file 2 — Additional file 2: eFigure 2 Covariate balance analysis comparing laparoscopic cholecystectomy (LC) to open cholecystectomy (OC). [file 13017_2023_521_MOESM2_ESM.jpg]

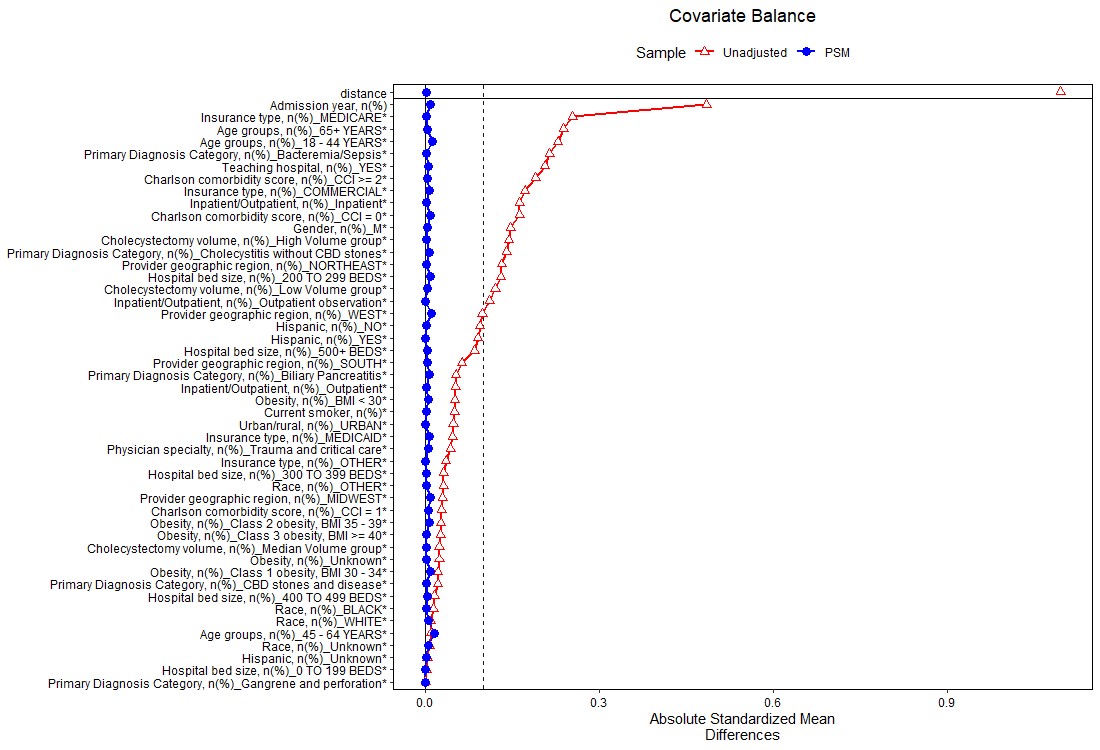

Supplement: Supplementary file 3 — Additional file 3: eFigure 3 Covariate balance analysis comparing robotic-assisted cholecystectomy (RAC) with open cholecystectomy (OC). [file 13017_2023_521_MOESM3_ESM.jpg]

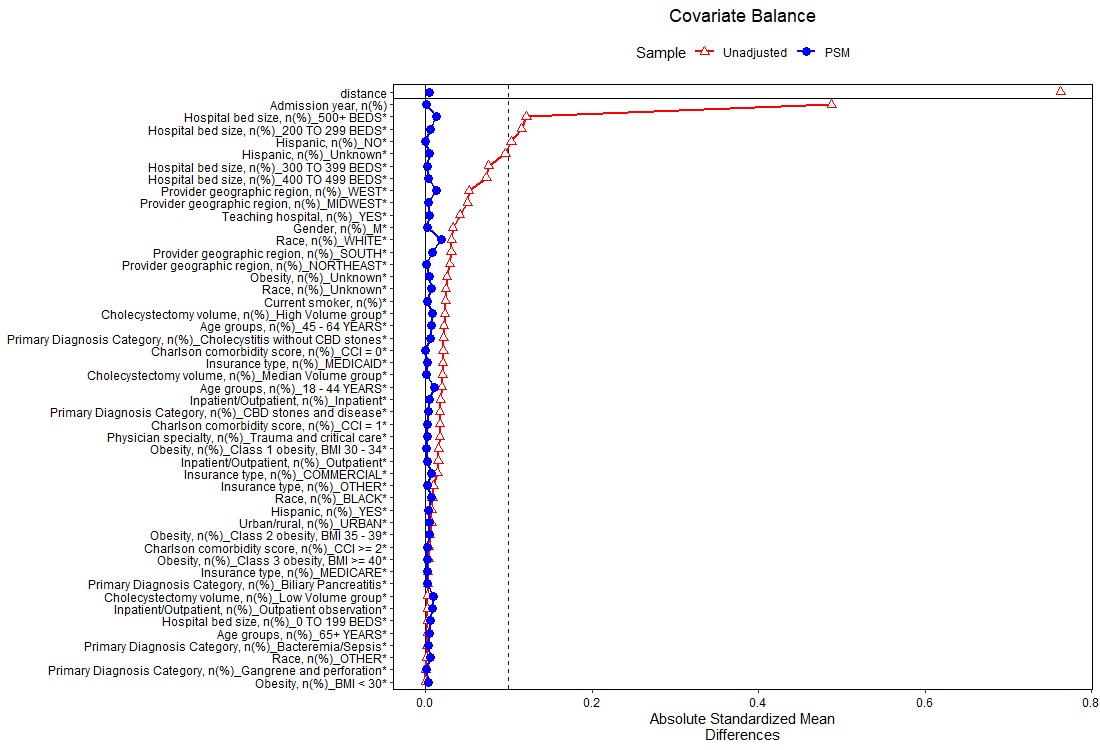

Supplement: Supplementary file 4 — Additional file 4: eFigure 4 Covariate balance analysis comparing robotic-assisted cholecystectomy (RAC) with fluorescent imaging vs RAC without any cholangiogram or fluorescent imaging. [file 13017_2023_521_MOESM4_ESM.jpg]

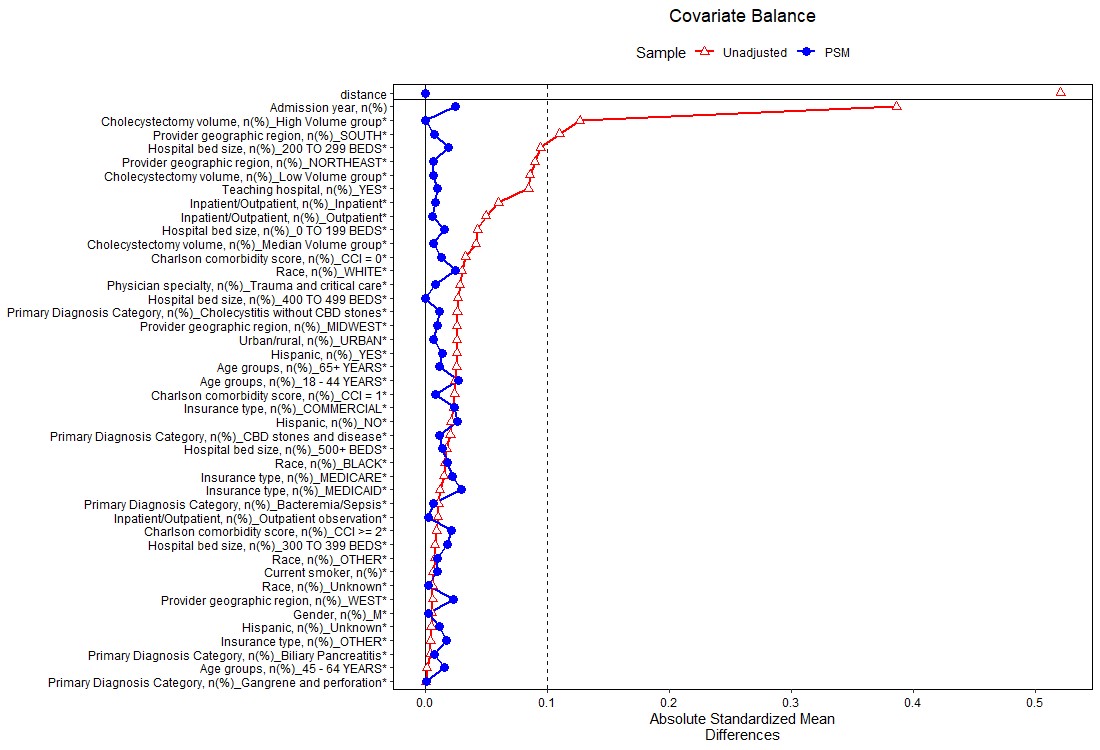

Supplement: Supplementary file 5 — Additional file 5: eFigure 5 Covariate balance analysis comparing robotic-assisted cholecystectomy (RAC) and laparoscopic cholecystectomy (LC) in patients with class 3 obesity. [file 13017_2023_521_MOESM5_ESM.jpg]
