# Supplementary material for: A retrospective study of laparoscopic, robotic-assisted, and open emergent/urgent cholecystectomy based on the PINC AI Healthcare Database 2017–2020
Source: World J Emerg Surg. 2023 Nov 30;18:55. doi: 10.1186/s13017-023-00521-8 (PMC10687827; doi:10.1186/s13017-023-00521-8)
Supplement: Supplementary file 6 — Additional file 6: eTable 1 Diagnosis and Procedure Codes Used to Identify Cases. [file 13017_2023_521_MOESM6_ESM.docx]

eTable 1. Diagnosis and Procedure Codes Used to Identify Cases

| **Variables** | **Diagnosis and procedure codes by code types** | |
| --- | --- | --- |
|  | **ICD - 10** | **CPT/HCPCS^a^** |
| Cholecystectomy | 0FT40ZZ, 0FT44ZZ, 0FB40ZZ, 0FB44ZZ as primary procedure code | 47562, 47563, 47564, 47600, 47605, 47610, 47612, 47620 |
| Surgical approach |  |  |
| Laparoscopic | 0FT44ZZ, 0FB44ZZ | 47562, 47563, 47564 |
| Robotic | 0FT44ZZ, 0FB44ZZ  + presence of following codes:  8E0W0CZ, 8E0W3CZ, 8E0W4CZ, 8E0W7CZ, 8E0W8CZ, 8E0WXCZ  Or + presence of robotic supplies in hospital billing | 47562, 47563, 47564  + presence of robotic HCPCS code S2900 |
| Open | 0FT40ZZ, 0FB40ZZ | 47600, 47605, 47610, 47612, 47620 |
| Primary diagnosis codes for hospital stay |  |  |
| Gangrene and perforation | K82.A1, K82.A2 | - |
| CBD stones and disease | K80.42, K80.43, K80.46, K80.47, K80.62, K80.63, K80.66, K80.67,  K80.30, K80.31, K80.32, K80.33, K80.34, K80.35, K80.36, K80.37, K80.40, K80.41, K80.44, K80.45, K80.50, K80.51, K80.60, K80.61, K80.64, K80.65, K80.70, K80.71 | - |
| Cholecystitis without CBD stones | K80.00, K80.01, K80.12, K80.13, K81.0,  K81.2, K80.12, K80.10, K80.11, K80.18,  K80.19, K80.20, K80.21, K81.9, K81.1,  K80.80, K80.81 | - |
| Biliary Pancreatitis | K85.10, K85.11, K85.12 | - |
| Sepsis and Bacteremia | A02.1, A32.7, A40.0, A40.1, A40.3, A40.8, A40.9, A41.01, A41.02, A41.1, A41.2, A41.3, A41.4, A41.50, A41.51, A41.52, A41.53, A41.59, A41.81, A41.89, A41.9, B37.7, R65.20, R78.81 | - |

^a^ HCPCS= Healthcare Common Procedure Coding System
