# Supplementary material for: A retrospective study of laparoscopic, robotic-assisted, and open emergent/urgent cholecystectomy based on the PINC AI Healthcare Database 2017–2020
Source: World J Emerg Surg. 2023 Nov 30;18:55. doi: 10.1186/s13017-023-00521-8 (PMC10687827; doi:10.1186/s13017-023-00521-8)
Supplement: Supplementary file 7 — Additional file 7: eTable 2 Diagnosis-Related Group (DRG) Codes Used for exclusion criteria. [file 13017_2023_521_MOESM7_ESM.docx]

eTable 2. Diagnosis Related Group (DRG) Codes Used for exclusion criteria

| **DRG Codes** | **Description** |
| --- | --- |
| 411 | Cholecystectomy With Common Duct Exploration (C.D.E) With Major Complication Or Comorbidity (Mcc) |
| 412 | Cholecystectomy With C.D.E. With Complication Or Comorbidity (Cc) |
| 413 | Cholecystectomy With C.D.E. Without Complication Or Comorbidity (Cc)/Major Complication Or Comorbidity (Mcc) |
| 414 | Cholecystectomy Except By Laparoscope Without C.D.E. With Major Complication Or Comorbidity (Mcc) |
| 415 | Cholecystectomy Except By Laparoscope Without C.D.E. With Complication Or Comorbidity (Cc) |
| 416 | Cholecystectomy Except By Laparoscope Without C.D.E. Without Complication Or Comorbidity (Cc)/Major Complication Or Comorbidity (Mcc) |
| 417 | Laparoscopic Cholecystectomy Without C.D.E. With Major Complication Or Comorbidity (Mcc) |
| 418 | Laparoscopic Cholecystectomy Without C.D.E. With Complication Or Comorbidity (Cc) |
| 419 | Laparoscopic Cholecystectomy Without C.D.E. Without Complication Or Comorbidity (Cc)/Major Complication Or Comorbidity (Mcc) |
| 853 | Infectious And Parasitic Diseases With O.R. Procedure With Major Complication Or Comorbidity (Mcc) |
| 854 | Infectious And Parasitic Diseases With O.R. Procedure With Complication Or Comorbidity (Cc) |
| 855 | Infectious And Parasitic Diseases With O.R. Procedure Without Complication Or Comorbidity (Cc)/Major Complication Or Comorbidity (Mcc) |
