# Supplementary material for: A retrospective study of laparoscopic, robotic-assisted, and open emergent/urgent cholecystectomy based on the PINC AI Healthcare Database 2017–2020
Source: World J Emerg Surg. 2023 Nov 30;18:55. doi: 10.1186/s13017-023-00521-8 (PMC10687827; doi:10.1186/s13017-023-00521-8)
Supplement: Supplementary file 8 — Additional file 8: eTable 3 Complications code list. [file 13017_2023_521_MOESM8_ESM.docx]

eTable 3. Complications code list

| **Complications** | **ICD – 10 codes** |
| --- | --- |
| Intraoperative complications | D78.12, E36.12, G97.48, I97.52, K91.71, K91.72, L76.12, M96.821, N99.71, N99.72, S25.401A, S31.001A, S31.609A, S35.219A, S35.221A, S35.222A, S35.228A, S35.229A, S35.239A, S35.299A, S35.319A, S35.329A, S35.331A, S35.338A, S35.339A, S35.349A, S35.403A, S35.404A, S35.405A, S35.406A, S35.511A, S35.512A, S35.513A, S35.514A, S35.515A, S35.516A, S35.531A, S35.532A, S35.533A, S35.534A, S35.535A, S35.536A, S35.8X9A, S35.90XA, S36.00XA, S36.020A, S36.021A, S36.029A, S36.030A, S36.031A, S36.032A, S36.039A, S36.09XA, S36.118A, S36.119A, S36.200A, S36.201A, S36.202A, S36.209A, S36.30XA, S36.400A, S36.408A, S36.409A, S36.500A, S36.501A, S36.502A, S36.503A, S36.508A, S36.509A, S36.60XA, S36.899A, S36.90XA, S37.001A, S37.002A, S37.009A, S37.029A, S37.10XA, S37.20XA, S37.30XA, T28.2XXA, T28.49XA, T28.7XXA, T28.99XA, T81.509A, T81.599A, T81.60XA, T81.61XA, T81.69XA, T81.89XA, T88.8XXA, Y65.8 |
| Postoperative complications | Any of gastrointestinal and digestive complications, pulmonary complications, cardiovascular complications, genitourinary complications, Neurological complications, or wound/infection complications |
| Conversion to open surgery | Z53.31, Z53.32, Z53.39 |
| Blood transfusion | 30233H0,30233H1,30233N0,30233N1, 30233P1,30243H0,30243H1,30243N0,30243N1,  30243P1,30253H0,30253H1,30253N0,30253N1,  30253P1, 30263H0,30263H1,30263N0,30263N1,30263P1,30263P0 |
| Sepsis | A40.0, A40.1, A40.3, A40.8, A40.9, A41.01, A41.02, A41.1, A41.2  A41.3, A41.4, A41.50, A41.51, A41.52, A41.53, A41.59, A41.81  A41.89, A41.9, A42.7, R65.10, R65.20, R65.21, R78.81 T81.12XA, T81.12XD, T81.12XS |
| **Gastrointestinal and digestive complications** | |
| Bile Duct Injury | S36.13XA, S36.129A, K83.3, K83.1 |
| Hepaticojejunostomy | 0F15 – 0F19, 0FQ5 - 0FQ9 |
| Intestinal Obstruction | K56.0 - K56.7 |
| Gastrointestinal ulcer | K25.0 – K25.9, K26.0 – K26.9, K27.0 – K27.9, K28.0 – K28.9 |
| Gastrointestinal hemorrhage | K92.0, K92.1, K92.2 |
| Retained Gallstone | K91.86 |
| **Pulmonary complications** | |
| Pneumonia/bronchitis/abscess of lung/air leak/respiratory failure | J12.0 – J12.9 , J13, J14, J15.0 – J15.9, J16.0, J16.8 , J18.0 – J18.9, J20.0 – J20.9, J69.0, J81.0, J85.2, J85.3, J86.0, J86.9, J90, J93.0, J93.11, J93.12, J93.82, J93.83, J95.3, J95.811, J95.812, J95.821, J95.822, J95.851, J95.859, J95.88, J95.89, J96.00, J96.20, J96.21, J96.22, J98.01, J98.11, J98.4, J98.51, R09.2 |
| Ventilator use >= 24 hrs | 5A09457, 5A09557, 5A1945Z, 5A1955Z |
| **Cardiovascular complications** | |
| Cardiac arrest | I45.0 – I45.9 |
| Congestive heart failure | I50.1 – I50.9 |
| Angina/Myocardial infarction | I20.0 – I20.9, I21.0 – I21.A, I22.0 – I22.9, I24.0 – I24.9 |
| Venous thromboembolism | I80.0 – I80.9, I82.0 – I82.9, T81.718A, T81.718D, T81.718S, T81.72XA, T81.72XD, T81.72XS |
| Portal vein thrombosis | I81 |
| **Genitourinary complications** | |
| Acute renal failure | N17.0 – N17.9 |
| Urinary tract infection | R34, R35.0 – R35.8, R39.0 – R39.9, N39.0 – N39.9 |
| **Neurological complications** | |
| Stroke/CVA | I66.0 – I66.9, G45.0 – G45.9, I67.89 |
| **Wound/infection complications** | |
| Surgical site infection | L03.319, L03.818, L03.90, L08.89, L08.9, T81.40XA, T81.40XD, T81.40XS, T81.41XA, T81.41XD, T81.41XS, T81.42XA, T81.42XD, T81.42XS, T81.43XA, T81.43XD, T81.43XS, T81.49XA, T81.49XD, T81.49XS, T81.4XXA, T81.4XXD, T81.4XXS |
| Hemorrhage/hematoma/seroma | D78.02, D78.22, D78.32, E36.02, E89.821, G97.32, G97.52, G97.62, I97.42, I97.618, I97.62, I97.620, I97.621, I97.638, J95.62, J95.831, J95.861, K91.62, K91.841, K91.871, L76.02, L76.22, L76.32, M96.811, M96.831, M96.841, N99.61, N99.62, N99.820, N99.821, N99.840, N99.841, D78.34, E89.823, G97.63, G97.64, H59.351, I97.622, J95.863, K91.873, L76.34, M70.98, M96.843, N99.842, N99.843, T79.2XXA, T88.8XXA |
| Wound disruption/dehiscence | T81.30 – T81.33, T81.40 – T81.49, K65 – K69 |
| Drainage of intraperitoneal abscess | 0D9U00Z, 0D9U0ZZ, 0D9V00Z, 0D9V0ZZ, 0D9W00Z, 0D9W0ZZ, 0W9G00Z, 0W9G0ZZ, 0WCJ0ZZ, 0WCP0ZZ, 0WCR0ZZ |
