# Supplementary material for: A retrospective study of laparoscopic, robotic-assisted, and open emergent/urgent cholecystectomy based on the PINC AI Healthcare Database 2017–2020
Source: World J Emerg Surg. 2023 Nov 30;18:55. doi: 10.1186/s13017-023-00521-8 (PMC10687827; doi:10.1186/s13017-023-00521-8)
Supplement: Supplementary file 9 — Additional file 9: eTable 4 Subgroup analyses post-propensity score matching (PSM): outcomes following robotic cholecystectomy without vs with fluorescent imaging. [file 13017_2023_521_MOESM9_ESM.docx]

**eTable 4.** Subgroup analyses post propensity score matching (PSM): outcomes following robotic cholecystectomy without vs with fluorescent imaging.

| **Fluorescence Subanalysis** | | | |
| --- | --- | --- | --- |
| **Parameter** | Without fluorescent imaging and IOC  (n = 3267) | With fluorescent imaging  (n = 3267) | p-value |
| **Index hospitalization outcomes** |  |  |  |
| Intraoperative complications, n (%) | 5 (0.2) | 12 (0.4) | 0.10 |
| Postoperative complications, n (%) | 368 (11) | 391 (12) | 0.46 |
| Conversion, n (%) | 74 (2.2) | 59 (1.8) | 0.27 |
| Blood transfusion, n (%) | 66 (2.0) | 63 (1.9) | 0.89 |
| Sepsis, n (%) | 108 (3.3) | 129 (3.9) | 0.25 |
| Gastrointestinal and digestive complications, n (%) |  |  |  |
| Bile duct injury | 6 (0.25) | 7 (0.2) | 0.87 |
| Retained gallstone | 1 (<0.15) | 0 | > 0.9 |
| Intestinal obstruction | 62 (1.9) | 66 (2.0) | 0.77 |
| Gastrointestinal ulcer | 2 (< 0.1) | 5 (0.25) | 0.33 |
| Gastrointestinal hemorrhage | 11 (0.35) | 7 (0.25) | 0.30 |
| Hospital length of stay, d |  |  | 0.42 |
| Mean (SD) | 3.7 (3.5) | 3.6 (3.5) |  |
| Median (IQR) | 3 (1,5) | 3 (1,4) |  |
| Operating room time, min |  |  | < 0.001 |
| Mean (SD) | 123.7 (52.9) | 134.5 (62.5) |  |
| Median (IQR) | 120 (90,150) | 120 (90,150) |  |
|  |  |  |  |
| **30-day Postoperative outcomes** |  |  |  |
| Sepsis, n (%) | 140 (4.3) | 152 (4.7) | 0.51 |
| Gastrointestinal and digestive complications, n (%) |  |  |  |
| Bile duct injury | 12 (0.4) | 10 (0.3) | 0.77 |
| Retained gallstone | 3 (<0.1) | 0 (0) | > 0.99 |
| Intestinal obstruction | 65 (2.0) | 67 (2.1) | 0.93 |
| Gastrointestinal ulcer | 6 (0.2) | 10 (0.3) | 0.32 |
| Gastrointestinal hemorrhage | 20 (0.6) | 14 (0.4) | 0.36 |
| Wound infection/complications, n (%) |  |  |  |
| Surgical site infection | 25 (0.8) | 26 (0.8) | 0.90 |
| Hemorrhage/hematoma/seroma | 9 (0.3) | 10 (0.3) | 0.81 |
| Wound disruption/dehiscence | 51 (1.6) | 42 (1.3) | 0.31 |
| Drainage of intraperitoneal abscess | 4 (0.1) | 4 (0.1) | > 0.99 |
| 30-day readmission, n (%) | 197 (6.0) | 167 (5.1) | 0.11 |
| 30-day reoperation, n (%) | 40 (1.2) | 31 (0.9) | 0.32 |

RAC = Robotic assisted cholecystectomy
