# Supplementary material for: A retrospective study of laparoscopic, robotic-assisted, and open emergent/urgent cholecystectomy based on the PINC AI Healthcare Database 2017–2020
Source: World J Emerg Surg. 2023 Nov 30;18:55. doi: 10.1186/s13017-023-00521-8 (PMC10687827; doi:10.1186/s13017-023-00521-8)
Supplement: Supplementary file 10 — Additional file 10: eTable 5 Subgroup analyses post-propensity score matching (PSM): outcomes following robotic and laparoscopic cholecystectomy on patients with class III obesity. [file 13017_2023_521_MOESM10_ESM.docx]

**eTable 5.** Subgroup analyses post propensity score matching (PSM): outcomes following robotic and laparoscopic cholecystectomy on patients with class III obesity.

| **Class III Obesity Subanalysis** | | | |
| --- | --- | --- | --- |
| **Parameter** | RAC  (n=1133) | Laparoscopic  (n=1133) | p-value |
| **Index hospitalization outcomes** |  |  |  |
| Intraoperative complications, n (%) | 7 (0.6) | 2 (0.2) | 0.12 |
| Postoperative complications, n (%) | 122 (11.0) | 121 (11.0) | > 0.99 |
| Conversion, n (%) | 30 (2.6) | 50 (4.4) | 0.02 |
| Blood transfusion, n (%) | 18 (1.6) | 20 (1.8) | 0.72 |
| Sepsis, n (%) | 32 (2.8) | 33 (2.9) | 0.92 |
| Gastrointestinal and digestive complications, n (%) |  |  |  |
| Bile duct injury | 2 (0.2) | 5 (0.4) | 0.38 |
| Retained gallstone | 0 (0.0) | 3 (0.3) | > 0.99 |
| Intestinal obstruction | 13 (1.1) | 16 (1.4) | 0.68 |
| Gastrointestinal ulcer | 0 (0.0) | 1 (< 0.1) | > 0.99 |
| Gastrointestinal hemorrhage | 2 (0.2) | 2 (0.2) | > 0.99 |
| Hospital length of stay, d |  |  | 0.31 |
| Mean (SD) | 3.6 (3.5) | 3.8 (3.8) |  |
| Median (IQR) | 3 (1,4) | 3 (1,5) |  |
| Operating room time, min |  |  | < 0.001 |
| Mean (SD) | 142.5 (63.3) | 125.1 (59.1) |  |
| Median (IQR) | 120 (101,172) | 110 (90, 150) |  |
|  |  |  |  |
| **30-day Postoperative outcomes** |  |  |  |
| Sepsis, n (%) | 40 (3.5) | 44 (3.9) | 0.71 |
| Gastrointestinal and digestive complications, n (%) |  |  |  |
| Bile duct injury | 2 (0.2) | 6 (0.5) | 0.20 |
| Retained gallstone | 0 (0.0) | 3 (0.3) | > 0.9 |
| Intestinal obstruction | 16 (1.4) | 13 (1.1) | 0.60 |
| Gastrointestinal ulcer | 4 (0.4) | 0 (0.0) | > 0.9 |
| Gastrointestinal hemorrhage | 4 (0.4) | 4 (0.4) | > 0.9 |
| Wound infection/complications, n (%) |  |  |  |
| Surgical site infection | 10 (0.9) | 10 (0.9) | >0.9 |
| Hemorrhage/hematoma/seroma | 6 (0.5) | 3 (0.3) | 0.32 |
| Wound disruption/dehiscence | 19 (1.7) | 14 (1.2) | 0.46 |
| Drainage of intraperitoneal abscess | 3 (0.3) | 1 (<0.1) | 0.36 |
| 30-day readmission, n (%) | 57 (5.0) | 50 (4.4) | 0.54 |
| 30-day reoperation, n (%) | 10 (0.9) | 11 (1.0) | 0.84 |

RAC = Robotic assisted cholecystectomy
