# Supplementary material for: A retrospective study of laparoscopic, robotic-assisted, and open emergent/urgent cholecystectomy based on the PINC AI Healthcare Database 2017–2020
Source: World J Emerg Surg. 2023 Nov 30;18:55. doi: 10.1186/s13017-023-00521-8 (PMC10687827; doi:10.1186/s13017-023-00521-8)
Supplement: Supplementary file 11 — Additional file 11: eTable 6 Multivariate analysis to identify risk factors for bile duct injury. [file 13017_2023_521_MOESM11_ESM.docx]

**eTable 6.** Multivariate analysis to identify risk factors for bile duct injury

| **Risk factors** | Odds ratio | 95% Confidence interval | p-value |
| --- | --- | --- | --- |
| Surgical modality |  |  |  |
| Laparoscopic | baseline |  |  |
| Robotic | 1.17 | (0.84 – 1.64) | 0.36 |
| Open | 2.15 | (1.44 – 3.21) | <0.001 |
| Age groups |  |  |  |
| 18–44 years | baseline |  |  |
| 45–64 years | 1.18 | (0.96 – 1.42) | 0.11 |
| 65+ years | 1.30 | (0.98 – 1.72) | 0.07 |
| Sex, n (%) |  |  |  |
| Female | baseline |  |  |
| Male | 1.22 | (1.06 – 1.41) | 0.01 |
| Obesity, n (%) |  |  |  |
| BMI 30–34, kg/m^2^ | 0.86 | (0.66 – 1.12) | 0.25 |
| BMI 35–39, kg/m^2^ | 0.90 | (0.68 – 1.19) | 0.46 |
| BMI ≥ 40, kg/m^2^ | 0.88 | (0.68 – 1.12) | 0.28 |
| Ethnicity, n (%) |  |  |  |
| Not Hispanic or Latino | baseline |  |  |
| Hispanic or Latino | 0.81 | (0.64 – 1.03) | 0.08 |
| Unknown | 0.89 | (0.74 – 1.08) | 0.25 |
| Race, n (%) |  |  |  |
| Caucasian | baseline |  |  |
| Black | 0.95 | (0.74 – 1.22) | 0.68 |
| Other | 0.90 | (0.67 – 1.10) | 0.23 |
| Unknown | 1.00 | (0.72 – 1.29) | 0.81 |
| Primary Diagnosis Category, n (%) |  |  |  |
| Cholecystitis w/o CBD stones | baseline |  |  |
| Biliary pancreatitis | 1.43 | (1.10 – 1.85) | 0.01 |
| Bacteremia/sepsis | 1.75 | (1.37 – 2.24) | 0.00 |
| CBD stones and disease | 3.94 | (3.38 – 4.60) | <0.001 |
| Gangrene and perforation | 4.72 | (0.64 – 33.44) | 0.13 |
| Charlson comorbidity score, n (%) |  |  |  |
| CCI = 0 | baseline |  |  |
| CCI = 1 | 0.91 | (0.73 – 1.12) | 0.37 |
| CCI ≥ 2 | 0.92 | (0.67 – 1.24) | 0.57 |
| Census region, n (%) |  |  |  |
| South | baseline |  |  |
| Midwest | 1.23 | (1.02 – 1.47) | 0.03 |
| Northeast | 0.90 | (0.71 – 1.13) | 0.36 |
| West | 1.15 | (0.95 – 1.40) | 0.15 |
| Admission year, n (%) |  |  |  |
| 2017 | baseline |  |  |
| 2018 | 0.84 | (0.71 – 1.01) | 0.06 |
| 2019 | 0.80 | (0.67 – 0.96) | 0.01 |
| 2020 | 0.50 | (0.41 – 0.63) | <0.001 |
| Admission type, n (%) |  |  |  |
| Inpatient | baseline |  |  |
| Outpatient | 0.47 | (0.24 – 0.92) | 0.03 |
| Outpatient observation < 24 hrs. | 0.90 | (0.69 – 1.20) | 0.51 |
| Hospital size, n (%) |  |  |  |
| 500+ beds | baseline |  |  |
| 0–199 beds | 0.80 | (0.63 – 1.00) | 0.05 |
| 200–299 beds | 0.81 | (0.65 – 1.01) | 0.06 |
| 300–399 beds | 0.95 | (0.77 – 1.17) | 0.62 |
| 400–499 beds | 1.01 | (0.81 – 1.25) | 0.95 |
| Physician Specialty, n (%) |  |  |  |
| General and colorectal surgery | baseline |  |  |
| Trauma and critical care surgery | 1.14 | (0.87 – 1.17) | 0.34 |
| Cholecystectomy volume 1 year prior to index surgery, n (%) |  |  |  |
| Medium volume group ^b^ | baseline |  |  |
| Low volume group ^c^ | 0.92 | (0.78 – 1.08) | 0.31 |
| High volume group ^a^ | 1.00 | (0.86 – 1.18) | 0.95 |

RAC = robotic-assisted cholecystectomy; BMI = body mass index; CCI= Charlson comorbidity index

^a^ Median number of procedures = 105

^b^ Median number of procedures = 45

^c^ Median number of procedures = 10
