# Supplementary material for: A retrospective study of laparoscopic, robotic-assisted, and open emergent/urgent cholecystectomy based on the PINC AI Healthcare Database 2017–2020
Source: World J Emerg Surg. 2023 Nov 30;18:55. doi: 10.1186/s13017-023-00521-8 (PMC10687827; doi:10.1186/s13017-023-00521-8)
Supplement: Supplementary file 12 — Additional file 12: eTable 7 Multivariate analysis to identify risk factors for conversion. [file 13017_2023_521_MOESM12_ESM.docx]

**eTable 7.** Multivariate analysis to identify risk factors for conversion

| **Risk factors** | Odds ratio | 95% Confidence interval | p-value |
| --- | --- | --- | --- |
| Surgical modality |  |  |  |
| Laparoscopic | baseline |  |  |
| Robotic | 0.54 | (0.47 – 0.63) | <0.001 |
| Age groups |  |  |  |
| 18–44 years | baseline |  |  |
| 45–64 years | 1.68 | (1.57 – 1.81) | <0.001 |
| 65+ years | 1.82 | (1.66 – 2.01) | <0.001 |
| Sex, n (%) |  |  |  |
| Female | baseline |  |  |
| Male | 1.74 | (1.67 – 1.83) | <0.001 |
| Obesity, n (%) |  |  |  |
| BMI 30–34, kg/m^2^ | 1.10 | (1.02 – 1.21) | 0.00 |
| BMI 35–39, kg/m^2^ | 1.27 | (1.17 – 1.39) | <0.001 |
| BMI ≥ 40, kg/m^2^ | 1.60 | (1.50 – 1.74) | <0.001 |
| Ethnicity, n (%) |  |  |  |
| Not Hispanic or Latino | baseline |  |  |
| Hispanic or Latino | 0.87 | (0.81 – 0.95) | <0.001 |
| Unknown | 1.07 | (1.01 – 1.15) | 0.02 |
| Race, n (%) |  |  |  |
| Caucasian | baseline |  |  |
| Black | 1.13 | (1.05 – 1.22) | <0.001 |
| Other | 0.92 | (0.85 – 1.00) | 0.06 |
| Unknown | 0.87 | (0.79 – 0.97) | 0.01 |
| Primary Diagnosis Category, n (%) |  |  |  |
| Cholecystitis w/o CBD stones | baseline |  |  |
| Biliary pancreatitis | 0.42 | (0.38 – 0.47) | <0.001 |
| Bacteremia/sepsis | 1.57 | (1.48 – 1.67) | <0.001 |
| CBD stones and disease | 0.87 | (0.82 – 0.92) | <0.001 |
| Gangrene and perforation | 1.96 | (0.89 – 4.31) | 0.09 |
| Charlson comorbidity score, n (%) |  |  |  |
| CCI = 0 | baseline |  |  |
| CCI = 1 | 1.10 | (1.03 – 1.18) | 0.01 |
| CCI ≥ 2 | 1.22 | (1.12 – 1.34) | <0.001 |
| Census region, n (%) |  |  |  |
| South | baseline |  |  |
| Midwest | 1.03 | (0.97 – 1.11) | 0.33 |
| Northeast | 0.94 | (0.87 – 1.00) | 0.07 |
| West | 0.86 | (0.80 – 0.92) | <0.001 |
| Admission year, n (%) |  |  |  |
| 2017 | baseline |  |  |
| 2018 | 0.89 | (0.84 – 0.94) | <0.001 |
| 2019 | 0.88 | (0.83 – 0.94) | <0.001 |
| 2020 | 0.80 | (0.75 – 0.86) | <0.001 |
| Admission type, n (%) |  |  |  |
| Inpatient | baseline |  |  |
| Outpatient | 0.03 | (0.01 – 0.07) | <0.001 |
| Outpatient observation < 24 hrs. | 0.02 | (0.01 – 0.04) | <0.001 |
| Hospital size, n (%) |  |  |  |
| 500+ beds | baseline |  |  |
| 0–199 beds | 0.89 | (0.82 – 0.86) | 0.00 |
| 200–299 beds | 0.93 | (0.86 – 1.00) | 0.06 |
| 300–399 beds | 1.09 | (1.02 – 1.18) | 0.01 |
| 400–499 beds | 1.29 | (1.20 – 1.39) | 0.00 |
| Physician Specialty, n (%) |  |  |  |
| General and colorectal surgery | baseline |  |  |
| Trauma and critical care surgery | 1.57 | (1.44 – 1.72) | <.001 |
| Cholecystectomy volume 1 year prior to index surgery, n (%) |  |  |  |
| Medium volume group ^b^ | baseline |  |  |
| Low volume group ^c^ | 1.09 | (1.04 – 1.15) | 0.00 |
| High volume group ^a^ | 0.77 | (0.73 – 0.82) | <0.001 |

RAC = robotic-assisted cholecystectomy; BMI = body mass index; CCI= Charlson comorbidity index

^a^ Median number of procedures = 105

^b^ Median number of procedures = 45

^c^ Median number of procedures = 10
