# Supplementary material for: A retrospective study of laparoscopic, robotic-assisted, and open emergent/urgent cholecystectomy based on the PINC AI Healthcare Database 2017–2020
Source: World J Emerg Surg. 2023 Nov 30;18:55. doi: 10.1186/s13017-023-00521-8 (PMC10687827; doi:10.1186/s13017-023-00521-8)
Supplement: Supplementary file 13 — Additional file 13: eTable 8 Multivariate analysis to identify risk factors for postoperative complication. [file 13017_2023_521_MOESM13_ESM.docx]

**eTable 8.** Multivariate analysis to identify risk factors for postoperative complication

| **Risk factors** | Odds ratio | 95% Confidence interval | p-value |
| --- | --- | --- | --- |
| Surgical modality |  |  |  |
| Laparoscopic | baseline |  |  |
| Robotic | 1.07 | (1.00 – 1.14) | 0.06 |
| Open | 2.41 | (2.19 – 2.65) | <0.001 |
| Age groups |  |  |  |
| 18–44 years | baseline |  |  |
| 45–64 years | 1.38 | (1.32 – 1.45) | <0.001 |
| 65+ years | 1.86 | (1.75 – 1.98) | <0.001 |
| Sex, n (%) |  |  |  |
| Female | baseline |  |  |
| Male | 1.20 | (1.16 – 1.24) | <0.001 |
| Obesity, n (%) |  |  |  |
| BMI 30–34, kg/m^2^ | 1.15 | (1.10 – 1.222) | <0.001 |
| BMI 35–39, kg/m^2^ | 1.10 | (1.05 – 1.18) | <0.001 |
| BMI ≥ 40, kg/m^2^ | 1.23 | (1.18 – 1.30) | <0.001 |
| Ethnicity, n (%) |  |  |  |
| Not Hispanic or Latino | baseline |  |  |
| Hispanic or Latino | 0.94 | (0.90 – 0.99) | 0.03 |
| Unknown | 1.14 | (1.10 – 1.19) | <0.001 |
| Race, n (%) |  |  |  |
| Caucasian | baseline |  |  |
| Black | 0.99 | (0.95 – 1.05) | 0.89 |
| Other | 0.96 | (0.91 – 1.01) | 0.16 |
| Unknown | 0.92 | (0.86 – 0.98) | 0.01 |
| Primary Diagnosis Category, n (%) |  |  |  |
| Cholecystitis w/o CBD stones | baseline |  |  |
| Biliary pancreatitis | 1.18 | (1.12 – 1.25) | <0.001 |
| Bacteremia/sepsis | 6.44 | (6.20 – 6.69) | <0.001 |
| CBD stones and disease | 1.10 | (1.06 – 1.15) | <0.001 |
| Gangrene and perforation | 2.49 | (1.52 – 4.08) | <0.001 |
| Charlson comorbidity score, n (%) |  |  |  |
| CCI = 0 | baseline |  |  |
| CCI = 1 | 1.33 | (1.28 – 1.39) | <0.001 |
| CCI ≥ 2 | 1.76 | (1.67 – 1.87) | <0.001 |
| Census region, n (%) |  |  |  |
| South | baseline |  |  |
| Midwest | 1.03 | (0.99 – 1.08) | 0.10 |
| Northeast | 0.87 | (0.83 – 0.92) | 0.00 |
| West | 1.06 | (1.02 – 1.11) | 0.00 |
| Admission year, n (%) |  |  |  |
| 2017 | baseline |  |  |
| 2018 | 0.98 | (0.94 – 1.02) | 0.27 |
| 2019 | 0.94 | (0.90 – 0.98) | 0.00 |
| 2020 | 0.95 | (0.91 – 0.99) | 0.02 |
| Admission type, n (%) |  |  |  |
| Inpatient | baseline |  |  |
| Outpatient | 1.21 | (1.09 – 1.35) | 0.01 |
| Outpatient observation < 24 hrs. | 1.95 | (1.86 – 2.06) | <0.001 |
| Hospital size, n (%) |  |  |  |
| 500+ beds | baseline |  |  |
| 0–199 beds | 0.97 | (0.97 – 1.05) | 0.28 |
| 200–299 beds | 0.96 | (0.93 – 1.02) | 0.06 |
| 300–399 beds | 0.97 | (0.91 – 1.00) | 0.16 |
| 400–499 beds | 0.86 | (0.82 – 0.91) | 0.00 |
| Physician Specialty, n (%) |  |  |  |
| General and colorectal surgery | baseline |  |  |
| Trauma and critical care surgery | 1.08 | (1.01 – 1.16) | 0.02 |
| Cholecystectomy volume 1 year prior to index surgery, n (%) |  |  |  |
| Medium volume group ^b^ | baseline |  |  |
| Low volume group ^c^ | 0.99 | (0.96 – 1.03) | 0.62 |
| High volume group ^a^ | 1.01 | (0.98 – 1.05) | 0.45 |

RAC = robotic-assisted cholecystectomy; BMI = body mass index; CCI= Charlson comorbidity index

^a^ Median number of procedures = 105

^b^ Median number of procedures = 45

^c^ Median number of procedures = 10
